# Automatic and Fast Encoding of Representational Uncertainty Underlies the Distortion of Relative Frequency

**DOI:** 10.1523/JNEUROSCI.2006-20.2021

**Published:** 2021-04-21

**Authors:** Xiangjuan Ren, Huan Luo, Hang Zhang

**Affiliations:** ^1^School of Psychological and Cognitive Sciences and Beijing Key Laboratory of Behavior and Mental Health, Peking University, Beijing, 100871, China; ^2^Academy for Advanced Interdisciplinary Studies, Peking University, Beijing, 100871, China; ^3^Peking-Tsinghua Center for Life Sciences, Peking University, Beijing, 100871, China; ^4^PKU-IDG/McGovern Institute for Brain Research, Peking University, Beijing, 100871, China; ^5^Key Laboratory of Machine Perception, Ministry of Education, Peking University, Beijing, 100871, China; ^6^Chinese Institute for Brain Research, Beijing, 100871, China

**Keywords:** decision making, judgment, MEG, probability distortion, steady-state response, time-resolved decoding

## Abstract

Humans do not have an accurate representation of probability information in the environment but distort it in a surprisingly stereotyped way (“probability distortion”), as shown in a wide range of judgment and decision-making tasks. Many theories hypothesize that humans automatically compensate for the uncertainty inherent in probability information (“representational uncertainty”) and probability distortion is a consequence of uncertainty compensation. Here we examined whether and how the representational uncertainty of probability is quantified in the human brain and its relevance to probability distortion behavior. Human subjects (13 female and 9 male) kept tracking the relative frequency of one color of dot in a sequence of dot arrays while their brain activity was recorded by MEG. We found converging evidence from both neural entrainment and time-resolved decoding analysis that a mathematically derived measure of representational uncertainty is automatically computed in the brain, despite it is not explicitly required by the task. In particular, the encodings of relative frequency and its representational uncertainty, respectively, occur at latencies of ∼300 and 400 ms. The relative strength of the brain responses to these two quantities correlates with the probability distortion behavior. The automatic and fast encoding of the representational uncertainty provides neural basis for the uncertainty compensation hypothesis of probability distortion. More generally, since representational uncertainty is closely related to confidence estimation, our findings exemplify how confidence might emerge before perceptual judgment.

**SIGNIFICANCE STATEMENT** Human perception of probabilities and relative frequencies can be markedly distorted, which is a potential source of disastrous decisions. But the brain is not just ignorant of probability; probability distortions are highly patterned and similar across different tasks. Recent theoretical work suggests that probability distortions arise from the brain's compensation of its own uncertainty in representing probability. Is such representational uncertainty really computed in the brain? To answer this question, we asked human subjects to track an ongoing stimulus sequence of relative frequencies and recorded their brain responses using MEG. Indeed, we found that the neural encoding of representational uncertainty accompanies that of relative frequency, although the former is not explicitly required by the task.

## Introduction

Humans do not have an accurate representation of probability or relative frequency information in the environment but distort it in a surprisingly stereotyped way. Typically, small probability is overestimated and large probability underestimated, which can be well fit by a linear-in-log-odds (LLO) model with two parameters (for review, see Zhang and Maloney, 2012). Such “probability distortion” phenomena occur in a variety of judgment and decision-making tasks, such as relative frequency estimation (Attneave, 1953; Lichtenstein et al., 1978; Varey et al., 1990), confidence rating (Gigerenzer et al., 1991; Erev et al., 1994; Wallsten et al., 1997), decision under risk (Tversky and Kahneman, 1992; Gonzalez and Wu, 1999; Luce, 2000; Zhang et al., 2020), and are also widely reported in animal behaviors (Yang and Shadlen, 2007; Stauffer et al., 2015; Constantinople et al., 2019; Ferrari-Toniolo et al., 2019). However, the neural computations that accompany such probability distortions remain largely unknown.

One computation that is assumed to be central to probability distortion (Martins, 2006; See et al., 2006; Fennell and Baddeley, 2012; Zhang et al., 2020) is compensation for the uncertainty inherent in the representation of noisy probability information (“representational uncertainty”). In particular, recent comparison among an extensive set of computational models of probability distortion (Zhang et al., 2020) suggests that, in their judgment and decision-making, people take into account representational uncertainty that is proportional to *p*(1 – *p*), where *p* denotes probability or relative frequency. That is, representational uncertainty is zero at *p* = 0 or 1 and maximal at *p* = 0.5. The *p*(1 – *p*) form had also been proved by Lebreton et al. (2015) as a measure of uncertainty that correlates with the explicitly reported confidence for a specific judged value. Specifically, their fMRI results showed that, during the valuation process, this uncertainty measure is automatically encoded in the ventromedial PFC, the brain region known for encoding value, even in the absence of explicit confidence rating. Motivated by previous neuroimaging studies and computational modeling, we ask whether representational uncertainty of probability information can be automatically encoded in human brain and, if yes, whether this encoding can precede the explicit judgment of probability, as theories of probability distortion would expect (Martins, 2006; See et al., 2006; Fennell and Baddeley, 2012; Zhang et al., 2020).

In the present study, we designed a new experimental paradigm where a major component of the representational uncertainty of probability information (i.e., *p*(1 – *p*); for details, see Results) was varied continuously over time. By using time-resolved neural measurements and assessing the temporal coupling between stimulus variables and ongoing brain activities, we examined whether and how the encoding of representational uncertainty proceeds in time and relates to probability distortion.

**Figure 1.**
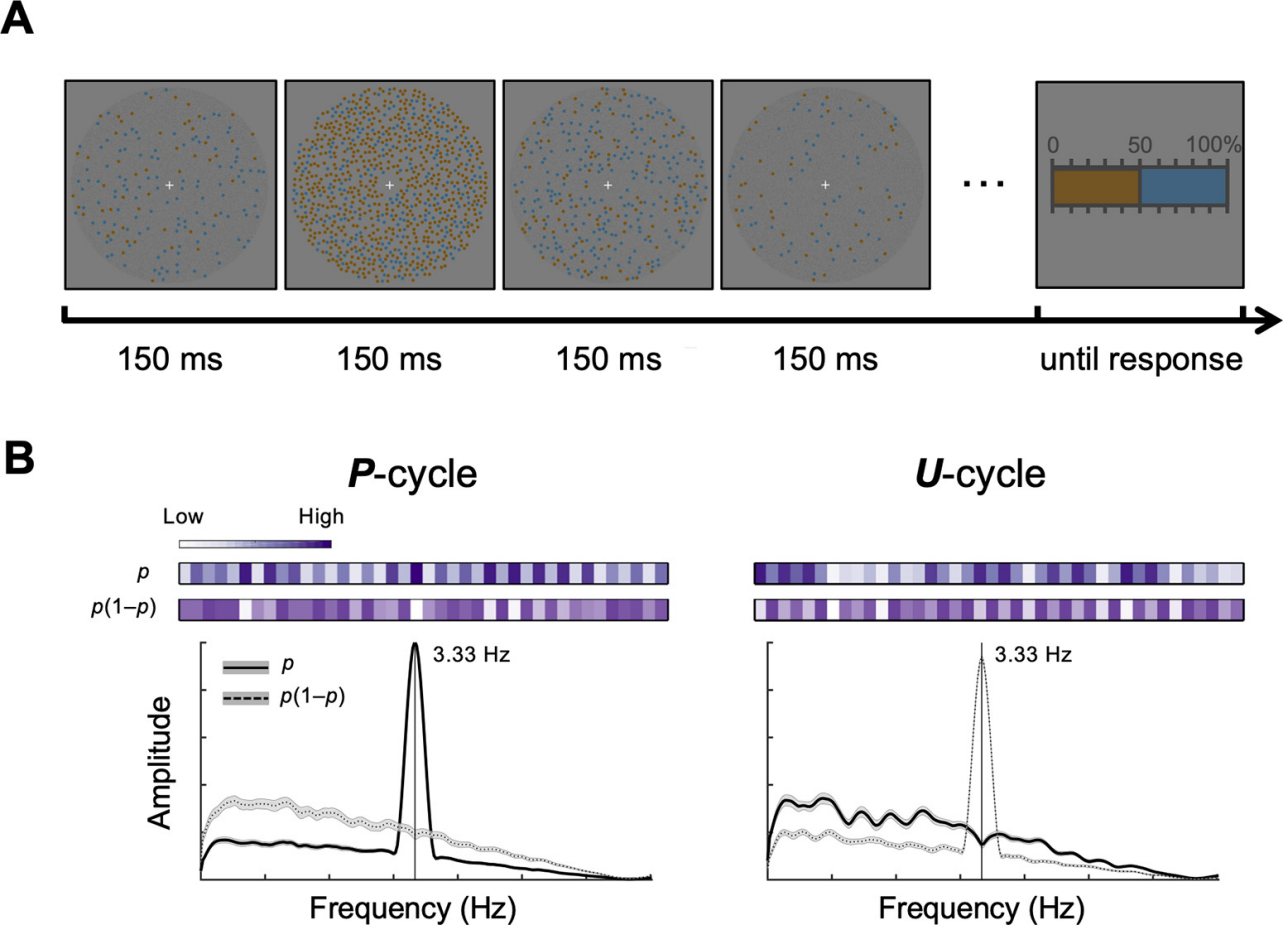
Experimental design. ***A***, Relative-frequency tracking and judgment task. On each trial, a sequence of orange and blue dot arrays was presented at a rate of 150 ms per display. Subjects were asked to fixate on the central fixation cross and track the relative frequency of orange (or blue) dots in each display until a 0%-100% response scale appeared. Subjects then clicked on the scale to report the relative frequency in the last display. ***B***, The *p* and *p*(1 – *p*) sequences in an example *P*-cycle or *U*-cycle trial. The value of *p* on each display was randomly chosen from a uniform distribution ranging from 0.1 to 0.9. In *P*-cycles (left panels), the *p* sequence was generated in such a way that the *p* value in different displays alternated between lower (<0.5) and higher (>0.5) values, resulting in a frequency spectrum peaking at 3.33 Hz (solid line), while the corresponding *p*(1 – *p*) sequence was aperiodic (dashed line). In *U*-cycles (right panels), it was the reverse: the *p*(1 – *p*) sequence alternated between lower (<0.21) and higher (>0.21) values in cycles of 3.33 Hz (dashed line), while the *p* sequence was aperiodic (solid line). To illustrate the periodicity or aperiodicity in the *p* and *p*(1 – *p*) sequences, the lower and higher *p* or *p*(1 – *p*) values are coded by lighter and darker colors, respectively, in the bars above the frequency spectrum plots. “lower” and “higher” do not mean discretization; the frequency spectra were computed from continuous values of *p* and *p*(1 – *p*).

Twenty-two human subjects participated in the study and were instructed to continuously track the relative frequency (*p*) of one color of dots in a sequence of dot arrays (see Fig. 1*A*), during which their brain activities were recorded by MEG. First, we found that, though *p* was the only variable that subjects needed to track while *p*(1 – *p*) was task-irrelevant, the periodic changes of *p*(1 – *p*) as well as *p* entrained neural rhythms, supporting an automatic tracking of representational uncertainty in the brain even when it is task-irrelevant. Next, by using a time-resolved decoding analysis to delineate the temporal course, we further found that the encoding of *p* and *p*(1 – *p*) peaked at ∼300 and 400 ms after stimulus onset, respectively. Finally, the relative strength of the neural responses to the two variables (*p* and *p*(1 – *p*)) in the frontoparietal region correlated with the variation of probability distortion behavior across individuals. Together, our results provide neural evidence for an automatic, fast encoding of representational uncertainty in the human brain that might underlie probability distortion observed in a wide range of human behaviors.

## Materials and Methods

### 

#### Experimental design

##### Subjects

Twenty-two human subjects (aged 18-27 years, 13 female) participated. No statistical methods were used to predetermine sample sizes, but our sample size was similar to that of previous human neuroimaging studies on probability or numerosity (Hsu et al., 2009; Lebreton et al., 2015; Fornaciai et al., 2017). All of them had normal or corrected-to-normal vision and passed the Farnsworth-Munsell 100 Hue Color Vision Test (Farnsworth, 1943). The study had been approved by the Institutional Review Board of School of Psychological and Cognitive Sciences at Peking University (#2015-03-13c). Subjects provided written informed consent in accordance with the Declaration of Helsinki and were compensated for their time.

##### Apparatus

Subjects were seated ∼86 cm in front of a projection screen (Panasonic PT-DS12KE: 49.6 × 37.2 cm, 1024 × 768 pixels, 60 Hz refresh rate) inside the magnetically shielded room. Stimuli were controlled by a Dell computer using MATLAB (The MathWorks) and PsychToolbox-3 (Brainard, 1997; Pelli, 1997). Subjects' behavioral responses were recorded by an MEG-compatible mouse system (FOM-2B-10B, Nata Technologies) and their brain activities by a 306-channel MEG system (for details, see MEG acquisition and preprocessing).

##### Task

Each trial started with a white fixation cross on a blank screen for 600 ms, following which a sequence of displays of orange and blue dots was presented on a gray background at a rate of 150 ms per display (see Fig. 1*A*). Subjects were asked to fixate on the central fixation cross and track the relative frequency of each display. After the sequence ended for 1000 ms, a horizontal scale (0%-100%) with the starting point in the middle of the bar appeared on the screen, and subjects were required to click on the scale to indicate the relative frequency of orange (or blue) dots on the last display. Half of the subjects reported relative frequency for orange dots and half for blue dots.

To encourage subjects to pay attention to each display, 1 of 6 trials were catch trials whose duration followed a truncated exponential distribution (1-6 s, mean 3 s), such that almost each display could be the last display. The duration of formal trials was 6 or 6.15 s. Only the formal trials were submitted to behavioral and MEG analyses.

On each display, all dots were randomly scattered without overlapping within an invisible circle that subtended a visual angle of 12°. The visual angle of each dot was 0.2°, and the center-to-center distance between any two dots was at least 0.12°. The two colors of the dots were isoluminant (CIE_orange_ = [43, 19.06, 52.33], CIE_blue_ = [43, –15.49, –23.72]). Isoluminant dark gray pixels (CIE_gray_ = [43, 0, 0]) were filled on the gray background (CIE_background_ = [56.5, 0, 0]) between the dots as needed to guarantee each display had equal overall luminance, which prevented luminance from being a confounding factor for any abstract variables of interest.

##### Design

We adopted a steady-state response (SSR) design, which could achieve a higher signal-to-noise ratio than the conventional event-related design (Norcia et al., 2015). The basic idea was to vary the value of a variable periodically at a specific temporal frequency and to observe the brain activities at the same frequency as an idiosyncratic response to the variable. The variables of most interest in the present study were relative frequency *p* and its representational uncertainty quantified by *p*(1 – *p*).

For half of the trials (referred as the *P*-cycle condition), the value of *p* in the sequence was chosen from uniform distributions with the ranges alternatively being (0.1, 0.5) and (0.5, 0.9) so that the sequence of *p* formed cycles of 3.33 Hz. For the other half trials (referred as the *U*-cycle condition), the value of *p* was chosen alternatively from (0.1, 0.3) U (0.7, 0.9) and (0.3, 0.7) so that the sequence of *p*(1 – *p*) (i.e., proxy for uncertainty) was in cycles of 3.33 Hz. For both the *P*-cycle and *U*-cycle conditions, *p* was randomly and independently sampled for each display within the defined distributions. That is, the two cycle conditions had matched individual displays but differed in which variable formed a periodic sequence. Accordingly, the *p*(1 – *p*) sequence in the *P*-cycle condition and the *p* sequence in the *U*-cycle condition were aperiodic, which had little autocorrelations.

The total number of dots on a display (numerosity, denoted *N*) was varied across trials as well as across individual displays of the same trial. The value of *N* for a specific display was independent of the value of *p* and generated as a linear transformation of a β random variable following Beta (0.1, 10) so that the number of dots in each color (*pN* or (1 – *p*)*N*), a possible confounding variable of *p*, would only have moderate correlations (Pearson's |*r|* < 0.5) with *p*. The linear transformation was chosen to locate *N* to the range of [10, 90] for half of the trials (the *N*-small condition) and to [100, 900] for the other half (the *N*-large condition).

The numbers of dots in each color was *pN* or (1 – *p*)*N* rounded to the nearest integer. As the result of rounding, the actual relative frequency was slightly different from the originally chosen *p*. In data analysis, we would use *p* to denote the actual relative frequency that was presented to subjects.

To summarize, there were 2 (*P*-cycle vs *U*-cycle) × 2 (*N*-small vs *N*-large) experimental conditions, with all conditions interleaved and each condition repeated for 6 trials in each block. Each subject completed 10 blocks of 24 trials, resulting in 50 formal trials and 10 catch trials for each condition. Before the formal experiment, there were 32 practice trials (the first 20 trials consisted of one display and the following 12 trials had the same settings as the formal experiment) for subjects to be familiar with the procedure. No feedback was available during the experiment.

#### Behavioral analysis

##### Measures of probability distortion

**Figure 2.**
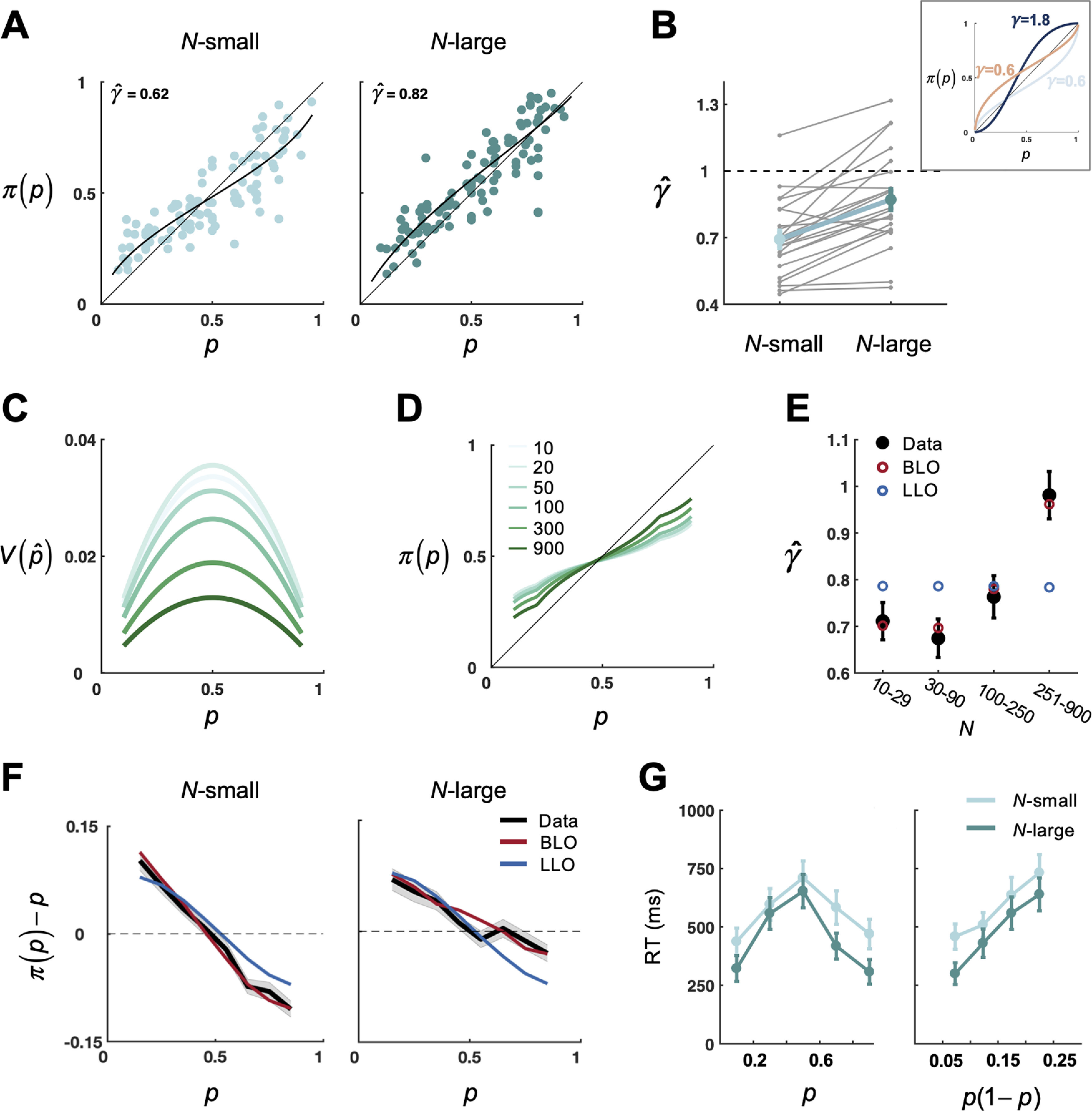
Behavioral and modeling results. ***A***, Probability distortion quantified by LLO model. Main plots, Distortion of relative frequency of 1 representative subject. The subject's reported relative frequency π(p)is plotted against objective relative frequency *p*, separately for the *N*-small and *N*-large conditions. Each dot represents one trial. Black curves indicate LLO model fits. ***B***, Slope of distortion $\hat{\gamma }$ for the *N*-small and *N*-large conditions. Gray line indicates 1 subject. Thick green line indicates the group mean. Error bars indicate SEM. Inset, Illustration of LLO distortion patterns with varying parameters γ and $p_{0}$. Gray blue, brown, and dark blue curves indicate [γ = 0.6, *p_0_* = 0.3], [γ = 0.6, *p_0_* = 0.7], and [γ = 1.8, *p_0_* = 0.3], respectively. ***C***, Illustration of representational uncertainty ($V\left(\hat{p}\right)$) as a function of *p* and *N*. Larger values of *N* are coded in darker green (same as in ***D***). The maximal sample size is assumed to follow the form $n_{s}=b+N^{a}$, with *a* = 0.42 and *b* = 1.91 (median estimates across subjects). ***D***, Illustration of probability distortion predicted by BLO: π(p) as a function of *p* and *N*. Larger values of *N* are coded in darker green. ***E***, Slopes of distortion: data versus model fits. According to the value of *N* in the last display, all trials were divided into four bins, and the slope of distortion, $\hat{\gamma }$, was estimated separately for each bin. Gray filled circles represent data. Error bars indicate SEM. Red and blue circles represent BLO and LLO model fits, respectively. ***F***, π(p)−p as a function of *p* and *N*. Black curves indicate smoothed data on the group level, separately for the *N*-small and *N*-large conditions. Shadings represent SEM. Red and blue curves represent BLO and LLO model fits, respectively. ***G***, RT as an additional index for representational uncertainty. The mean RT for subjects to initiate their relative frequency report is plotted against binned *p* or *p*(1 – *p*), separately for the *N*-small and *N*-large conditions. Error bars indicate SEM.

According to Zhang and Maloney (2012), inverted-*S*- or *S*-shaped probability distortions can be well captured by the LLO model as follows:

(1) $$\lambda \left[\pi \left(p\right)\right]=\gamma \lambda \left[p\right]+\left(1-\gamma \right)\lambda \left[p_{0}\right]+\varepsilon_{\lambda },$$

where p and π(p), respectively, denote the objective and subjective probability or relative frequency, $\lambda \left[p\right]=\log\frac{p}{1-p}$ is the log-odds transformation, and $\varepsilon_{\lambda }$ is Gaussian error on the log-odds scale with mean 0 and variance $\sigma_{\lambda }^{2}$. The free parameter γ is the slope of distortion, with γ<1 corresponding to inverted-*S*-shaped distortion, γ=1 to no distortion, and γ>1 to *S*-shaped distortion. The free parameter $p_{0}$ is the crossover point where π(p)=p. For three examples illustrating the functional form of the LLO model (Eq. 1), see Figure 2*B* (inset).

For each subject and condition, we fit the reported relative frequency to the LLO model (Eq. 1) and used the estimated $\hat{\gamma }$ and ${\hat{p}}_{0}$ as the measures of relative frequency distortions.

##### Response time (RT)

The RT was defined as the interval between the response screen onset and subjects' first mouse move. The RT of the first trial of 3 subjects was mis-recorded because of technical issues and was excluded from further analysis. We divided all trials evenly into 5 bins based on the to-be-reported *p*, and computed the mean *p* and mean RT for each bin. A similar binning procedure was applied to *p*(1 – *p*) to visualize the relationship of RT to *p*(1 – *p*).

##### Nonparametric measures of probability distortion

As a complement to the LLO model (Eq. 1), we used a nonparametric method to visualize the probability distortion curve for each subject. In particular, we smoothed π(p)−p using a kernel regression method with the commonly used Nadaraya-Watson kernel estimator (Nadaraya, 1964; Watson, 1964; Aljuhani and Al Turk, 2014) as follows:

(2) $${\hat{M}}_{h}\left(x\right)=\frac{\sum_{i=1}^{m}K\left(\frac{x-x_{i}}{h}\right)y_{i}}{\sum_{i=1}^{m}K\left(\frac{x-x_{i}}{h}\right)},$$

where $x_{i}$ and $y_{i}$ (i=1,2,...,m) denote observed pairs of stimuli and responses, ${\hat{M}}_{h}\left(x\right)$ denotes the smoothed response at the stimulus value x, and h is a parameter that controls the degree of smoothing and were set to be 0.03. The $K\left(\cdot \right)$ denotes the Gaussian kernel function as follows:

(3) $$K\left(z\right)=\frac{1}{\sqrt{2\pi }}\exp\left(-\frac{z^{2}}{2}\right).$$

#### Behavioral modeling

##### Bounded log-odds (BLO) model

Zhang et al. (2020) proposed the BLO model of probability distortion, which is based on three assumptions. Below, we briefly describe these assumptions and how they are applied to the present study.

1. Probability is internally represented in log-odds. BLO assumes that any probability or relative frequency, *p*, is internally represented as a linear transformation of log-odds, as follows:
  (4) $$\lambda \left(p\right)=\log\frac{p}{1-p},$$
  which potentially extends from minus infinity (λ(0) = –∞)to infinity (λ(1)=∞).
2. Representation is encoded on a bounded Thurstone scale.
  By “Thurstone scale,” we refer to a psychological scale perturbed by independent, identically distributed Gaussian noise, which was proposed by Thurstone (1927) and has been widely used in modeling representations of psychological magnitudes (e.g., Erev et al., 1994; Lebreton et al., 2015). BLO assumes that the Thurstone scale for encoding log-odds has a limited range [−Ψ,Ψ] and can only encode a selected interval of log-odds. It defines bounding operation as follows:
  (5) $$\Gamma \left[\lambda \right]=\{\begin{array}{ll} \Delta^{-}, & \lambda <\Delta^{-}\\ \lambda , & \Delta^{-}\leq \lambda \leq \Delta^{+}\\ \Delta^{+}, & \lambda >\Delta^{+} \end{array}$$
  to confine the representation of log-odds λ to the interval $\left[\Delta^{-},\Delta^{+}\right]$, where $\Delta^{-}$ and $\Delta^{+}$ are free parameters. That is, any λ(p) that is outside the bounds will be truncated to its nearest bound. The bounded interval $\left[\Delta^{-},\Delta^{+}\right]$ is mapped to the bounded Thurstone scale [−Ψ,Ψ] through a linear transformation, so that the log-odds λ(p) is encoded as follows:
  (6) $$\Lambda \left(p\right)=\eta \left[\Gamma \left(\lambda \left[p\right]\right)-\left(\Delta^{-}+\Delta^{+}\right)/2\right],$$
  where $\eta \equiv \frac{\Psi }{\left(\Delta^{+}-\Delta^{-}\right)/2}$ is a free parameter.
3. Representational uncertainty is compensated in the final estimate of probability. In our relative frequency judgment task, we assume representational uncertainty partly arises from random variation of sampling. Suppose subjects may not access all of the dots in a display. For a display of N dots, a sample of $n_{s}$ dots is randomly drawn from the display without replacement and used to infer the relative frequency. The resulting variance of $\hat{p}$ is as follows (Cochran, 1977; Zhang et al., 2020):
  (7) $$V\left(\hat{p}\right)\;=\frac{p\left(1-p\right)}{n_{s}}\frac{N-n_{s}}{N-1}.$$

In the present study, $n_{s}$ is modeled as an increasing function of numerosity as follows:

(8) $$n_{s}=b+N^{a},$$

where a≥0 and b≥0 are free parameters. To keep the resulting $V\left(\hat{p}\right)\geq 0$, we forced $V\left(\hat{p}\right)=0$ whenever $V\left(\hat{p}\right)<0$. Equation 7 does not necessarily imply that $V\left(\hat{p}\right)$ is an increasing function of *N*, given that $n_{s}$ is not constant but may vary with *N* (Eq. 8). Indeed, for a median subject (*a* = 0.42 and *b* = 1.91) in our experiment, $V\left(\hat{p}\right)$ is mostly a decreasing function of *N*.

BLO assumes that representational uncertainty is compensated in the final estimate of probability, with the log-odds of π(p) modeled as a weighted average of Λ(p) and a fixed point $\Lambda_{0}$ on the log-odds scale as follows:

(9) $$\lambda \left[\pi \left(p\right)\right]=\omega_{p}\Lambda \left(p\right)+\left(1-\omega_{p}\right)\Lambda_{0}+\varepsilon_{\lambda },$$

where $\omega_{p}=\frac{1}{1+\alpha V\left(\hat{p}\right)}$ is a measure of the reliability of the internal representation, and $\varepsilon_{\lambda }$ is Gaussian noise term on the log-odds scale with mean 0 and variance $\sigma_{\lambda }^{2}$. Here, α≥0 and $\sigma_{\lambda }>0$ are free parameters. In total, the BLO model for our relative frequency judgment task has eight parameters: $\Delta^{-}$, $\Delta^{+}$, a, b, η, $\Lambda_{0}$, α, and $\sigma_{\lambda }$.

##### Model fitting

We considered the BLO model and 17 alternative models (described below). For each subject, we fit each model to the subject's π(p) in each trial using maximum likelihood estimates. The *fminsearchbnd* (J. D'Errico), a function based on *fminsearch* in MATLAB (The MathWorks), was used to search for the parameters that minimized negative log likelihood. To verify that we had found the global minimum, we repeated the searching process for 1000 times with different starting points.

##### Factorial model comparison

Similar to Zhang et al. (2020), we performed a factorial model comparison (van den Berg et al., 2014) to test whether each of the three assumptions in the BLO model outperformed plausible alternative assumptions in fitting behavioral data. The models we considered differ in the following three dimensions.

*D1: scale of transformation*. The scale on which probability is internally represented can be the log-odds scale ($\lambda \left(p\right)=\log\frac{p}{1-p}$ as in Eq. 4), the Prelec scale ($\lambda^{\prime }\left(p\right)=-\log\left(-\log p\right)$) derived from the two-parameter Prelec function (Prelec, 1998), or the linear scale used in the neo-additive probability distortion functions (for review, see Wakker, 2010; for more details, see Zhang et al., 2020).

*D2: bounded versus bounds-free*. It concerns whether the bounding operation (Eq. 5) is involved.

*D3: variance compensation*. BLO compensates for representational uncertainty following Equation 9. In relative frequency judgment, the form of $V\left(\hat{p}\right)$ is not only proportional to *p*(1 – *p*) but also depends on *N* and sampling strategy. In the present study, we modeled sample size as $n_{s}=b+N^{a}$, which increases with *N*. Alternatively, sample size $n_{s}$ may be modeled as a constant. Under constant $n_{s}$, the value of $V\left(\hat{p}\right)$ is still proportional to *p*(1 – *p*). A third alternative assumption is to set $V\left(\hat{p}\right)=\text{constant}$, which is not theoretically motivated but effectively implemented in classic descriptive models of probability distortion, such as LLO or the Prelec function.

These three dimensions are independent of each other, analogous to the different factors manipulated in a factorial experimental design. In total, there are 3 (D1: log-odds, Prelec, or linear) × 2 (D2: bounded or bounds-free) × 3 (D3: $V\left(\hat{p}\right)$ with $n_{s}=b+N^{a}$, $V\left(\hat{p}\right)$ with constant $n_{s}$, or constant $V\left(\hat{p}\right)$) = 18 models.

In this three-dimensional model space, the BLO model we introduced earlier corresponds to (D1 = log-odds, D2 = bounded, D3=$V\left(\hat{p}\right)$ with $n_{s}=b+N^{a}$). The model at (D1 = log-odds, D2 = bounded, D3=$V\left(\hat{p}\right)$ with constant $n_{s}$) is also a BLO model, which differs from our main BLO model in the specification of sampling strategies. For simplicity, in presenting the results of factorial model comparison, we will only refer to our main BLO model as BLO. The LLO, and two-parameter Prelec models are also special cases of the 18 models, respectively, corresponding to (D1 = log-odds, D2 = bounds-free, D3 = constant $V\left(\hat{p}\right)$) and (D1 = Prelec, D2 = bounds-free, D3 = constant $V\left(\hat{p}\right)$).

##### Model comparison method: Akaike information criterion with a correction for sample sizes (AICc) and group-level Bayesian model selection

We compared the goodness of fit of the BLO model with all of the alternative models based on the AICc (Akaike, 1974; Hurvich and Tsai, 1989) and group-level Bayesian model selection (Stephan et al., 2009; Daunizeau et al., 2014; Rigoux et al., 2014).

##### Model comparison method: 10-fold cross-validation

We used a 10-fold cross-validation (Arlot and Celisse, 2010) to verify the model comparison results based on the metric of AICc. In particular, we divided all trials in each cycle and numerosity condition randomly into 10 folds. Each time 1 fold served as the test set and the remaining 9 folds as the training set. The model parameters estimated from the training set were applied to the test set, for which the log likelihood of the data was calculated. Such computation of cross-validated log likelihood was repeated for each fold as the test set. The cross-validated log likelihoods of all 10 folds were summed and multiplied by –2 to result in a metric comparable to AICc, denoted –2LL. For each subject, we used the model with the lowest –2LL as a reference to compute Δ(–2LL) for each model and summed Δ(–2LL) across subjects. For each subject, we repeated the above procedure 10 times to reduce randomness and reported the average summed cross-validated Δ(–2LL).

##### Model identifiability analysis

To evaluate potential model misidentification issues in model comparisons, we performed a further model identifiability analysis as follows. Model parameters had been estimated for individual subjects, and the model parameters estimated from the 22 subjects' real data were used to generate synthetic datasets of 22 virtual subjects. We generated 50 synthetic datasets for each of the 18 models considered in our factorial model comparison analysis, then fit all the 18 models to each synthetic dataset and identified the best fitting model. For each synthetic dataset, the model with the lowest summed ΔAICc was identified as the best fitting model. For the 50 datasets generated by each specific model, we calculated the percentages that the model was correctly identified as the best model and that each of the other models was mistakenly identified as the best model.

#### MEG acquisition and preprocessing

Subjects' brain activity was recorded by a 306-channel whole-head MEG system (Elekta-Neuromag, 102 magnetometers and 102 pairs of orthogonal planar gradiometers). Head position was measured before each block by an isotrack polhemus system with four head position indicator coils (two on the left and right mastoid, the other two on the left and right forehead below the hairline). Subjects whose between-block head movement exceeded 3 mm would be excluded from further analysis. Horizontal and vertical electro-oculograms were recorded to monitor eye-movement artifacts. Sampling rate was set to be 1000 Hz, and an analog bandpass filter from 0.1 to 330 Hz was applied. Maxwell filtering was used to minimize external magnetic interference and to compensate for head movements (Taulu and Kajola, 2005; Taulu and Simola, 2006).

Standard preprocessing procedures were applied using MATLAB 2016b and the FieldTrip package (Oostenveld et al., 2011). The MEG data of each block was first low-pass filtered <20 Hz and then segmented into epochs of 7.6 s relative to trial onset (–0.6 to 7 s). Independent component analysis was applied to aggregated epoch data to remove artifacts, including blinks, eye movements, breaths, and heart activity. No subject was excluded for excessive head movement or other artifacts. For 2 subjects, the first trial of one block was excluded because of the failure of synchronization between stimulus onset and MEG recording onset at the beginning of the block.

#### Phase coherence analysis

Given a periodically changed stimulus sequence (*p* in *P*-cycle or *p*(1 – *p*) in *U*-cycle), stimulus-evoked brain responses imply that the phase difference between the stimulus and brain response time series should be coherent across trials at the stimulus frequency (Summerfield and Mangels, 2005). The phase coherence across trials is defined (Kramer and Eden, 2016) as follows:

(10) $$\kappa =\frac{\left|{\hat{P}}_{rs}\right|}{\sqrt{{\hat{P}}_{ss}}\sqrt{{\hat{P}}_{rr}}},$$

where ${\hat{P}}_{ss}$ and ${\hat{P}}_{rr}$, respectively, denote the trial-averaged power spectrum for stimulus and brain response time series, and $\left|{\hat{P}}_{rs}\right|$ denotes the magnitude of the trial-averaged cross-spectrum between stimuli and responses. The value of κ for any specific frequency is between 0 and 1, with larger κ indicating stronger phase-locked responses.

For a specific variable (*p* or *p*(1 – *p*)), we computed the phase coherence between stimulus sequence and MEG signals separately for each subject, each magnetometer, and each cycle and numerosity condition. In particular, we downsampled epoched MEG time series to 300 Hz, applied zero padding and Hanning window to single trials, and used FFT to calculate power spectrum. The resulting frequency resolution was 0.03 Hz.

To evaluate the chance-level phase coherence, we shuffled the MEG time series across different time points within each trial and computed phase coherence for the permutated time series. This permutation procedure was repeated 500 times to produce a distribution of chance-level phase coherences.

#### Decoding analysis

##### Time-resolved decoding

For an aperiodic stimulus sequence (*p*(1 – *p*) in *P*-cycle or *p* in *U*-cycle), we could infer the brain's encoding of the stimulus variable at a specific time lag to stimulus onset by examining how well the value of the variable could be reconstructed from the neural responses at the time lag. In particular, we performed a time-resolved decoding analysis using regression methods (Lalor et al., 2006; Wyart et al., 2012; Gonçalves et al., 2014; Crosse et al., 2016) as follows:

(11) $$S\left(t\right)=\sum_{n}g\left(\tau ,n\right)R\left(t+\tau ,n\right)+\varepsilon \left(t\right),$$

where S(t) denotes the value of the stimulus that starts at time *t*, g(τ,n) denotes the to-be-estimated decoding weight for channel n at time lag τ, R(t+τ,n) denotes the response of channel n at time t+τ, and ε(t) is a Gaussian noise term.

We used the MEG signals of all 306 sensors (102 magnetometers and 204 gradiometers) at single time lags to decode *p* from *U*-cycle trials or *p*(1 – *p*) from *P*-cycle trials, separately for each subject, each cycle and numerosity condition, and each time lag between 0 and 900 ms. Epoched MEG time series were first downsampled to 120 Hz. To eliminate multicollinearity and reduce overfitting, we submitted the time series of the 306 sensors to a principal component analysis and used the first 30 components (explaining ∼95% variance) as the regressors. The decoding weights (i.e., regression coefficients) were estimated for normalized stimuli and responses using the L2-rectified regression method implemented in the mTRF toolbox (Crosse et al., 2016), with the ridge parameter set to 1.

We used a leave-one-out cross-validation (Arlot and Celisse, 2010) to evaluate the predictive power of decoding performance as the following. Of the 50 trials in question (or 49 trials in the case of trial exclusion), each time one trial served as the test set and the remaining trials as the training set. The decoding weights estimated from the training set were applied to the test set, for which Pearson's *r* was calculated between the predicted and the ground-truth stimulus sequence. Such computation was repeated for each trial as the test set, and the averaged Pearson's *r* was used as the measure for decoding performance.

We identified time windows that had above-chance decoding performance using cluster-based permutation tests (Maris and Oostenveld, 2007) as follows. Adjacent time lags with significantly positive decoding performance at the uncorrected significance level of 0.05 by right-sided one-sample *t* tests were grouped into clusters, and the summed *t* value across the time lags in a cluster was defined as the cluster-level statistic. We randomly shuffled the stimulus sequence of each trial, performed the time-resolved decoding analysis on the shuffled sequence, and recorded the maximum cluster-level statistic. This procedure was repeated 500 times to produce a reference distribution of chance-level maximum cluster-level statistic, based on which we calculated the *P* value for each cluster in real data. This test effectively controls the Type I error rate in situations involving multiple comparisons. For each subject, we defined the median of all time points for which the decoding performance exceeded the 95% percentile of the distribution as the time lag of the peak (Marti et al., 2015).

##### Spatial decoding

For the conditions and time windows that had above-chance performances in the time-resolved decoding analysis, we further performed a spatial decoding analysis at individual sensor location to locate the brain regions that are involved in encoding the variable in question. For a specific senor location, the MEG signals at its three sensors (one magnetometer and two gradiometers) within the time window were used for decoding. The time window that enclosed the peak in the time-resolved decoding was 125-ms-wide and 167-ms-wide, respectively, for *p* and *p*(1 – *p*) under *N*-large condition. The decoding procedure was similar to that of the time-resolved decoding described above, except that the number of principal component analysis component used was 19 and 24, respectively, for decoding *p* and *p*(1 – *p*), which explained ∼99% variance of the original 48 (16 time lags × 3 sensors) and 63 (21 time lags × 3 sensors) channels.

Considering that the magnetometer and the two planar gradiometers may be sensitive to very different locations, depths, and orientations of underlying dipoles, we also performed spatial decoding separately for magnetometers and pairs of gradiometers using all temporal samples within the decoded time windows, without any principal component analysis procedure.

The same cluster-based permutation test described above was applied to the spatial decoding results, except that the cluster-level test statistic was defined as the sum of the *t* values of the continuous sensors (separated by gaps < 4 cm) (Maris and Oostenveld, 2007; Groppe et al., 2011) in a given cluster.

#### Ruling out the effects of confounding factors

We considered the following eight variables in the stimuli as potential confounding factors, which may covary with *p* or *p*(1 – *p*) across displays.

1. *N*: the total number of dots (numerosity) in the display, which by design was independent of both *p* and *p*(1 – *p*).
2. *N_t_*: the number of dots in the target color in the display, that is, *N_t_* = *Np*.
3. *N_o_*: the number of dots in the other color in the display, that is, *N_o_* = *N*(1 – *p*).
4. *AvgLumi*: the mean luminance of the display, which was kept constant across different displays and different trials and was thus independent of both *p* and *p*(1 – *p*).
5. vCIE-L*: the color variance of the display on the L* dimension in the CIELAB color space, where L* ranges from black to white.
6. vCIE-a*: the color variance of the display on the a* dimension in the CIELAB color space, where a* ranges from green to red.
7. vCIE-b*: the color variance of the display on the b* dimension in the CIELAB color space, where b* ranges from blue to yellow.
8. M-contrast: the Michelson luminance contrast (Wiebel et al., 2016), that is, M-contrast = $\frac{L_{dots}-L_{background}}{L_{dots}+L_{background}}$.

According to our design, most of these variables had negligible correlations with *p* and *p*(1 – *p*), and none of the absolute values of correlation coefficients exceeded 0.53 (Pearson's *r*). The numerosity (*N*) had considerable correlations (Pearson's |*r*| > 0.79) with all other confounding factors, except for the mean luminance (*AvgLumi*), which was constant across displays. We performed similar phase coherence and time-resolved decoding analyses for these variables as we did for *p* and *p*(1 – *p*) but obtained very different patterns, which suggests that the findings we reported for *p* and *p*(1 – *p*) in the main text are unlikely to be effects of confounding factors. The patterns of all these confounding factors except for *AvgLumi* were almost identical, which may have the same origins, such as the automatic encoding of numerosity (Fornaciai et al., 2017; Park et al., 2016).

#### Time-resolved decoding analysis based on cross-validated version of confound regression (CVCR)

To further rule out the possibility that the observed automatic encoding of *p*(1 – *p*) may be an artifact of confounding factors, we performed a time-resolved decoding analysis based on the CVCR method proposed by Snoek et al. (2019), which allowed us to regress out confounding variables from MEG time series before decoding analysis.

The CVCR method consists of two modules: encoding and decoding. During encoding, we fit a multiple linear regression model to MEG time series separately for each magnetometer or gradiometer and each specific time lag, with the confounding variables as regressors as follows:

(12) $$Y_{j}\left(\tau \right)=\sum_{i=1}^{8}\beta_{j,i}\left(\tau \right)C_{i}+\beta_{j,0}+\varepsilon_{j}\left(\tau \right),$$

where *j* denotes sensor number, τ denotes time lag after stimulus onset (ranging from 0 to 900 ms), $Y_{j}\left(\tau \right)$ denotes MEG signals at sensor *j* and time lag τ, $C_{i}$ (*i* = 1, 2, …, 8) denotes the value of the *i*-th confounding variable, among the eight confounding variables we considered above (i.e., *N*, *N_t_*, *N_o_*, *AvgLumi*, vCIE-L*, vCIE-a*, vCIE-b*, and *M-contrast*), $\beta_{j,i}$ and $\beta_{j,0}$ are free parameters, and $\varepsilon_{j}\left(\tau \right)$ is a Gaussian noise term.

Then, subtracting the explained variance of confounding variables from MEG signals and defined the residual as “confounds-regressed-out MEG signal” as follows:

(13) $$Y_{j,reg}\left(\tau \right)=Y_{j}\left(\tau \right)-\left(\sum_{i=1}^{8}{\hat{\beta }}_{j,i}\left(\tau \right)C_{i}+{\hat{\beta }}_{j,0}\right).$$

The confounds-regressed-out MEG signal, $Y_{j,reg}\left(\tau \right)$, was subsequently used for time-resolved decoding analysis.

Following suggestions by Snoek et al. (2019), we used a leave-one-out procedure as follows. Of the 50 trials in question (or 49 trials in the case of trial exclusion), each time one trial served as the test set and remaining trials as the training set. The parameters (of Eq. 12) estimated within each fold of training data, ${\hat{\beta }}_{j,i}^{train}$ and ${\hat{\beta }}_{j,0}^{train}$, were used to remove the variance related to the confounds from both the training set and test set. The resulting confounds-regressed-out training data, $Y_{j,reg}^{train}$, and test data, $Y_{j,reg}^{test}$, were then concatenated and used for the decoding module.

#### Statistical analysis

##### Linear mixed models (LMMs)

LMMs were estimated using 'fitlme' function in MATLAB R2016b, whose *F* statistics, degree of freedom of residuals (denominators), and *P* values were approximated by the Satterthwaite method (Giesbrecht and Burns, 1985; Fai and Cornelius, 1996; Kuznetsova et al., 2017). Specifications of random effects in LMMs were kept as maximal as possible (Barr et al., 2013) but without overparameterizing (Bates et al., 2015). The LMMs reported in Results are described below.

*LMM1 and LMM2*: the fitted LLO parameters $\hat{\gamma }$ and ${\hat{p}}_{0}$ are, respectively, the dependent variables; fixed effects include an intercept, the main and interaction effects of categorical variables cycle condition (*P*-cycle vs *U*-cycle) and numerosity condition (*N*-small versus *N*-large); random effects include correlated random slopes of cycle and numerosity conditions within subjects and random subject intercept.

*LMM3 and LMM4*: the fitted LLO parameters $\hat{\gamma }$ and ${\hat{p}}_{0}$ are, respectively, the dependent variables; fixed effects include an intercept and the main effect of categorical variable numerosity bin (all trials were evenly divided into four bins according to the value of *N* in the last display); random effects include correlated random slopes of numerosity bin within subjects and random subject intercept.

*LMM5*: the logarithm of RT, log(RT), is the dependent variable; fixed effects include an intercept, the main effects of continuous variables *p*, *p*(1 – *p*) and *N*; random effects include correlated random slopes of *p*, *p*(1 – *p*) and *N* within subjects and random subject intercept.

##### Correction for multiple comparisons

For phase coherence or decoding performance, grand averages across subjects were computed and permutation tests described above were used to assess their statistical significance over chance (Maris and Oostenveld, 2007). The false discovery rate (*FDR*) method (Benjamini and Hochberg, 1995; Benjamini and Yekutieli, 2001) was used for multiple-comparison corrections whenever applicable. For the phase coherence spectrum averaged across magnetometers, *FDR* corrections were performed among the 235 frequency bins within 0-7 Hz. For the phase coherence topography at 3.33 Hz, *FDR* corrections were performed among all 102 magnetometers.

##### Difference between the peak latencies of p(1 – p) and p

We defined the peak latency of the decoding performance of *p* or *p*(1 – *p*) as follows. First, for the temporal course of the decoding performance of a specific variable, adjacent time points with above-chance *t* values (uncorrected right-sided *P* < 0.05 by one-sample *t* test) were grouped into clusters. Among clusters between 100 and 500 ms, we then identified the cluster with the largest sum of *t* values and defined the centroid position (i.e., *t* value-weighted average of the time points) of the largest cluster as the peak latency of the variable. For each subject, we estimated the peak latencies of *p* and *p*(1 – *p*) in their decoding performance and computed the latency difference between *p*(1 – *p*) and *p*.

To test whether the latency difference between *p*(1 – *p*) and *p* was statistically significant, we estimated the 95% CI of the difference using a bootstrap method (Efron and Tibshirani, 1993) as follows. In each virtual experiment, we randomly resampled 22 virtual subjects with replacement from the 22 subjects and calculated the mean latency difference across the virtual subjects. The virtual experiment was repeated 1000 times to produce a sampling distribution of the peak latency difference, based on which we estimated the 95% CI of the peak latency difference.

### Data availability

All data are available for download at https://osf.io/db4ms.

## Results

As shown in Figure 1*A*, on each trial, subjects saw a sequence of displays consisting of isoluminant orange and blue dots that was refreshed every 150 ms, and were instructed to track the relative frequency of dots in one color (i.e., orange or blue, counterbalanced across subjects). To ensure subjects' persistent tracking throughout the trial, the sequence of displays ended after random duration and subjects needed to report the relative frequency of the last display by clicking on a percentage scale afterward.

A 2 (*P*-cycle or *U*-cycle) × 2 (*N*-small or *N*-large) experimental design was used, as we describe below. Specifically, for *P*-cycle trials, the sequence of displays was generated according to the *p* value (randomly chosen from a uniform distribution between 0.1 and 0.9) and the displays alternated between lower values of *p* (<0.5) and higher values of *p* (>0.5), with every two displays forming a cycle. As the consequence, the values of *p* in *P*-cycle trials varied at a rhythm of 3.33 Hz, whereas the values of *p*(1 – *p*) would be aperiodic. In contrast, for *U*-cycle trials (*U* for uncertainty), the sequence of displays was generated according to the value of *p*(1 – *p*) in a similar way so that the displays alternated between lower values of *p*(1 – *p*) (<0.21) and higher values of *p*(1 – *p*) (>0.21) in cycles of 3.33 Hz, while the values of *p* were aperiodic. In other words, in *P*-cycle trials, the *p* value underwent a rhythmic fluctuation and the *p*(1 – *p*) value followed an aperiodic random course, whereas the opposite pattern occurred for *U*-cycle trials. Figure 1*B* illustrates the *P*-cycle (left) and *U*-cycle (right) conditions.

Our experimental design had two important features. First, the values of *p* and *p*(1 – *p*) were statistically independent of each other. Second, the values of *p* and *p*(1 – *p*) were dissociated in periodicity. The subjects' task was always to attend to *p* (relative frequency), while *p*(1 – *p*) was completely task-irrelevant in both *P*-cycle and *U*-cycle trials.

In addition, the total number of dots in a display (numerosity, denoted *N*) was not constant but varied from display to display, independently of *p* or *p*(1 – *p*), ranging from 10 to 90 (*N*-small trials) or from 100 to 900 (*N*-large trials). Manipulating *N* as well as *p* allowed us to manipulate the slope of probability distortion (Zhang and Maloney, 2012) and thus to further investigate the encoding of representational uncertainty (Schustek et al., 2019).

### Behavioral probability distortions quantified by LLO

All 22 subjects performed well on reporting the relative frequency of the last display, whose subjective estimate π(p) was highly correlated with the objective relative frequency *p* (all Pearson's *r* > 0.71, *P* < 0.001). Given that the stimulus sequence might end unexpectedly, if subjects had failed to track the sequence, they might have missed the last stimulus. However, the mean percentage of large errors (|π(p)−p|>0.4) was low, not only for the normal trials (0.7%) but also for the catch trials (1.9%), although the latter was a little higher than the former (paired-sample *t* test, *t*_(21)_ = 2.49, *P* = 0.021). Subjects' sensible reports thus provide evidence that they had tracked the change of *p* throughout the trial as instructed.

Figure 2*A* shows the reported π(p) of one representative subject, who showed a typical inverted-*S*-shaped probability distortion, overestimating small *p* and underestimating large *p*. As expected, most subjects exhibited the typical inverted-*S*-shaped probability distortion, and a few subjects exhibited the opposite *S*-shaped distortion, a profile also consistent with previous findings (Varey et al., 1990; Zhang and Maloney, 2012).

We next used the LLO model to quantify such inverted-*S*- or *S*-shaped distortions, summarizing each subject's probability distortion behavior for each cycle (*P*-cycle or *U*-cycle) and dot numerosity (*N*-small or *N*-large) conditions by two parameters fitted from π(p) (see Materials and Methods): slope $\hat{\gamma }$ and crossover point ${\hat{p}}_{0}$. We used linear mixed-effects model analyses (numbered in Materials and Methods) to identify the effects of cycle conditions (*P*-cycle vs *U*-cycle), numerosity conditions (*N*-small vs *N*-large), and their interactions on the estimated $\hat{\gamma }$ (LMM1) and ${\hat{p}}_{0}$ (LMM2). Only numerosity showed a significant influence on $\hat{\gamma }$ (*F*_(1,21.00)_ = 36.30, *P* < 0.001). No other main effects or interactions on $\hat{\gamma }$ or ${\hat{p}}_{0}$ reached significance (all *P* > 0.10). In particular, the $\hat{\gamma }$ estimated from *N*-large trials was greater than that of *N*-small trials by 25.75% (Fig. 2*B*). Thus, our subsequent analysis will mainly focus on $\hat{\gamma }$ and collapse the two cycle conditions to estimate $\hat{\gamma }$. As illustrated by the inset of Figure 2*B*, a greater $\hat{\gamma }$ implies a less curved inverted-*S*-shaped distortion (for $\hat{\gamma }$<1) or a more curved *S*-shaped distortion (for $\hat{\gamma }$>1).

It is noteworthy that LLO was only used as a way to quantify probability distortions and their differences between conditions. The LLO model by itself could not explain why the slope of distortion would differ between numerosity conditions, as observed here (Fig. 2*B*). In contrast, the observed different $\hat{\gamma }$ between the *N*-small and *N*-large conditions could be well captured by the BLO model (Zhang et al., 2020), which compensates for the *p*(1 – *p*) form of representational uncertainty, with greater $\hat{\gamma }$ implying lower representational uncertainty. Next, we present the BLO model and how it may account for our behavioral results. These modeling analyses provide behavioral evidence for the encoding of representational uncertainty in the brain, which motivates the subsequent MEG analysis. Readers who are not interested in behavioral modeling may feel free to skip some details of the modeling results.

### BLO model and its behavioral evidence

Zhang et al. (2020) proposed the BLO model as a rational account of probability distortion, which can explain why probability distortion may vary with task or individual. BLO has three main assumptions (for details, see Materials and Methods): (1) probability is internally represented as log-odds, (2) representation is truncated to a bounded scale, and (3) representational uncertainty is compensated in the final estimate of probability, so that estimates associated with higher uncertainty will be discounted to a greater extent. A full description of BLO would be out of the scope of the present article; we thus focus only on the uncertainty compensation assumption below.

According to BLO, when representational uncertainty (denoted $V\left(\hat{p}\right)$) is higher, the subjective estimate of probability is less contributed by the percept of the objective probability and more by a prior estimate, which implies that the higher the representational uncertainty, the shallower the slope of probability distortion. As illustrated in Figure 2*C*, the representational uncertainty modeled in our experiment (see Materials and Methods) is proportional to *p*(1 – *p*), that is, an inverted-*U*-shaped function of *p*. Meanwhile, for a median subject, $V\left(\hat{p}\right)$ first slightly increases with *N* (Fig. 2*C*, from *N* = 10 to *N* = 20) and then dramatically decreases with *N* (Fig. 2*C*, from *N* = 20 to *N* = 900). Consequently, BLO predicts that the slope of distortion $\hat{\gamma }$ is not necessarily constant, but may slightly decrease with *N* for very small *N* and mostly increase with larger *N* (Fig. 2*D*), which is indeed observed in our experiment (Fig. 2*E*). As expected, linear mixed-effect model analyses showed that the slope of distortion ($\hat{\gamma }$) increased with *N* (LMM3: *F*_(3,22.00)_ = 21.81, *p* < 0.001) and the crossover point of distortion (${\hat{p}}_{0}$) hardly varied with *N* (LMM4: *F*_(3,22.00)_ = 2.11, *P* = 0.13).

We are aware that representational uncertainty $V\left(\hat{p}\right)$ here is not equivalent but proportional to *p*(1 – *p*). In the neural analysis we will present, we chose to focus on the encoding of *p*(1 – *p*) (as a proxy for representational uncertainty) instead of $V\left(\hat{p}\right)$, because $V\left(\hat{p}\right)$ was highly correlated with numerosity *N* and thus would be difficult to be separated from the latter in brain activities. In contrast, *p*(1 – *p*) and *N* were independent of each other by design. In addition, *p*(1 – *p*) has a nice connection with the “second-order valuation” proposed by Lebreton et al. (2015).

#### Model versus data

Zhang et al. (2020) has provided behavioral evidence for the BLO model in two different tasks, including relative frequency judgment. Here we performed similar tests on our behavioral data and fit the BLO model to the reported π(p) for each subject using maximum likelihood estimates. The performance of the LLO model served as a baseline. When evaluating LLO's performance in fitting the data, we estimated one set of parameters for all conditions instead of estimating different parameters for different conditions as when we applied LLO as a measuring tool for probability distortions. In Figure 2*F*, we plot the observed π(p)−p (smoothed and averaged across subjects) as a function of *p* separately for different numerosity conditions and contrasted it with the BLO and LLO model predictions. The BLO prediction agreed well with the observed probability distortion function, while LLO largely failed.

We further examined how well BLO could predict the observed numerosity effect in the slope of distortion. For each subject, we divided the trials of each numerosity condition evenly into two bins according to the value of *N* in the last display (i.e., the display that was reported) and then estimated $\hat{\gamma }$ for each of the four bins. The pattern of $\hat{\gamma }$ in real data, initial slight decrease and subsequent dramatic increase with *N*, was quantitatively predicted by the fitted BLO model (Fig. 2*E*).

#### Model comparisons

**Figure 3.**
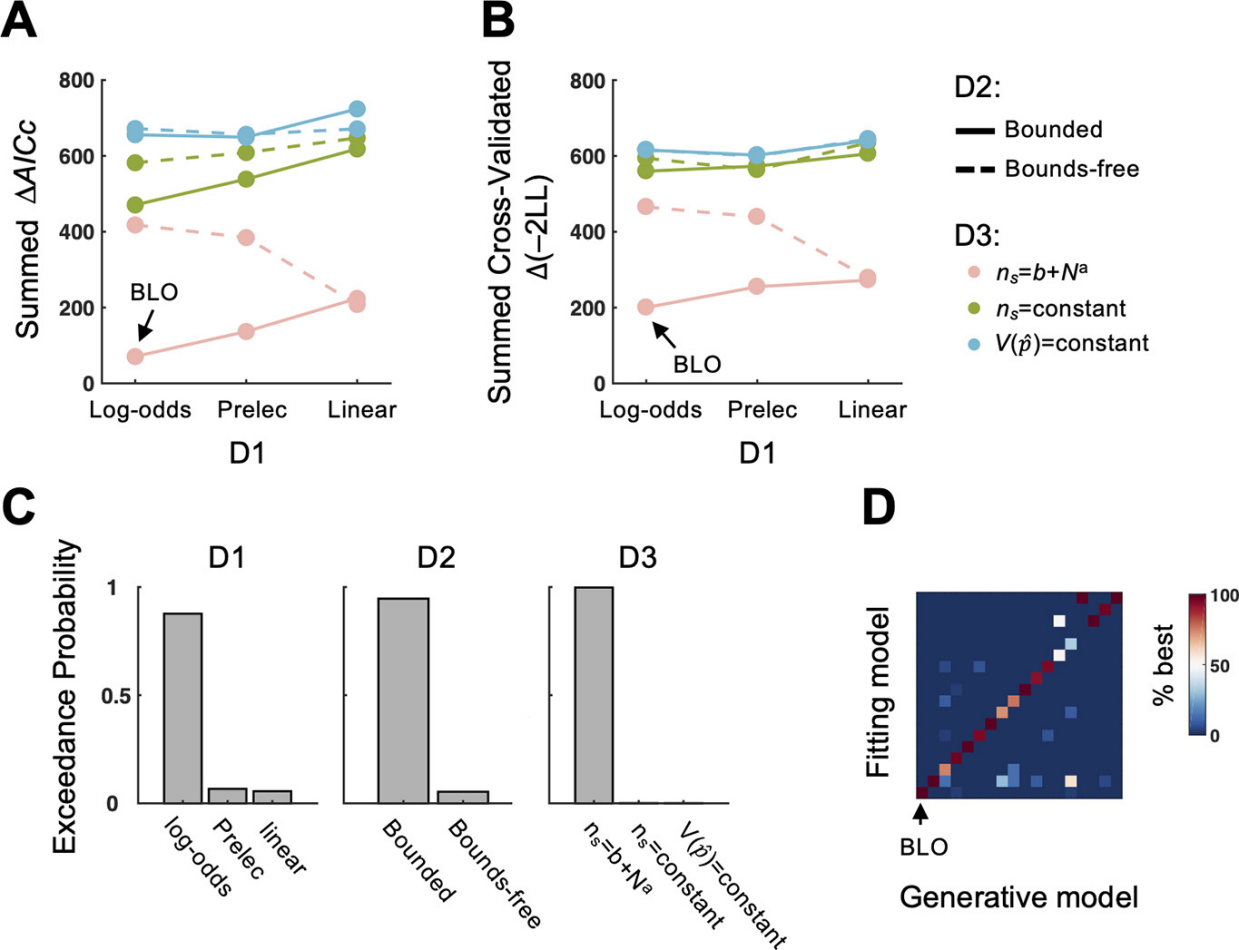
Results of factorial model comparisons. ***A***, Model comparison method: AICc. For each subject, the model with the lowest AICc was used as a reference to compute ΔAICc for each model. A lower value of ΔAICc summed across subjects indicates better fit. The BLO model outperformed all the alternative models. ***B***, Model comparison method: 10-fold cross-validation. The cross-validated log likelihood times −2 (denoted −2LL), which is comparable to AICc, was computed. For each subject, the model with the lowest −2LL was used as a reference to compute Δ(−2LL) for each model. Same as AICc, a lower value of Δ(−2LL) summed across subjects indicates better fit. Again, the BLO model outperformed all the alternative models. D1, D2, and D3 denote the three dimensions of factorial model comparison. ***C***, Protected exceedance probability on each model dimension. Each panel is for model comparison on one dimension. Each assumption of the BLO model outperformed the alternative assumptions on its dimension. ***D***, Model identifiability analysis. Each column is for one specific model that was used to generate synthetic datasets. Each row is for one model that was fitted to the synthetic datasets. Summed ΔAICc was used to identify the best fitting model for each dataset. The color of each cell codes the percentage that the model on its row was identified as the best model among the 50 datasets generated by the model on its column. Higher value is coded as more reddish and lower value as more bluish. Values in each column add up to 1. From left (bottom) to right (top), the 18 models are 111, 112, 113, 121, 122, 123, 211, 212, 213, 221, 222, 223, 311, 312, 313, 321, 322, and 323, where the first digit indexes the D1 assumption (1 for log-odds, 2 for Prelec, and 3 for linear), the second digit indexes the D2 assumption (1 for bounded and 2 for bounds-free), and the third digit indexes the D3 assumption (1 for $V\left(\hat{p}\right)$ with $n_{s}=b+N^{a}$, 2 for $V\left(\hat{p}\right)$ with constant $n_{s}$, and 3 for constant $V\left(\hat{p}\right)$). The BLO model with sample size $n_{s}=b+N^{a}$ is the first model (111), corresponding to the leftmost column and the bottom row, which is indicated by an arrow in the plot. The synthetic data that were generated by BLO were all best fit by BLO (see leftmost column), and those generated by the other models were seldom best fit by BLO (see bottom row).

Similar to Zhang et al. (2020), we performed a factorial model comparison (van den Berg et al., 2014) to test whether each of the three assumptions in the BLO model outperformed plausible alternative assumptions in fitting behavioral data. Accordingly, we constructed 3 × 3 × 2 = 18 models and fit each model for each subject using maximum likelihood estimates (for details, see Materials and Methods). The AICc (Akaike, 1974; Hurvich and Tsai, 1989), which punishes overfitting from additional free parameters, was used as the metric of goodness of fit. Lower AICc indicates better fit. For each subject, the model with lowest AICc was used as a reference to compute ΔAICc for each model. According to the summed ΔAICc across subjects, BLO was the best model among the 18 models (Fig. 3*A*). We further verified the superiority of BLO using a 10-fold cross-validation analysis, which reached the same conclusion (Fig. 3*B*).

We also evaluated whether each of BLO's three assumptions was the best among its alternatives on the same dimension. In particular, we used the group-level Bayesian model selection (Stephan et al., 2009; Daunizeau et al., 2014; Rigoux et al., 2014) to compute the probability that each specific model outperforms the other models in the model set (“protected exceedance probability”) and marginalized the protected exceedance probabilities for each dimension (i.e., adding up the protected exceedance probabilities across the other two dimensions). Indeed, all three assumptions of the BLO model in the present study — log-odd, bounded, and compensation for $V\left(\hat{p}\right)$ (with $n_{s}=b+N^{a}$) — outperformed their alternatives with probabilities of 87.7%, 94.6%, and 99.8%, respectively (Fig. 3*C*).

Furthermore, we performed a model recovery analysis (similar to Correa et al., 2018) to confirm that the advantage of BLO in factorial model comparison is real and does not result from model misidentification. In particular, we generated 50 synthetic datasets for each of the 18 models. All the datasets generated from BLO were best fit by BLO. Of the 850 datasets generated from the other models, only 0.24% were misidentified to BLO (Fig. 3*D*).

We also verified that the parameters of BLO could be reasonably well identified. Even if some of the BLO parameters were not perfectly identified, it would not influence the results of the neural analysis we report below, most of which did not rely on the estimated BLO parameters.

### Evidence for representational uncertainty in RT

The above modeling analysis on subjects' reported relative frequency (i.e., model fits as well as the factorial model comparison) suggests that subjects might compensate for representational uncertainty that is proportional to *p*(1 – *p*) and the *N*-large condition was associated with lower uncertainty than the *N*-small condition.

The RT of reporting relative frequency (defined as the interval between the response screen onset and the first mouse move) provides an additional index for representational uncertainty, given that lower uncertainty would lead to shorter RTs. Consistent with Lebreton et al. (2015), RTs were longest at *p* = 0.5 and shortest when *p* was close to 0 or 1 (Fig. 2*G*, left). More precisely, RTs increased almost linearly with *p*(1 – *p*) (Fig. 2*G*, right), in agreement with what one would expect if representational uncertainty is proportional to *p*(1 – *p*). In addition, RTs were shorter in *N*-large trials than in *N*-small trials, which echoes the lower $V\left(\hat{p}\right)$ for larger *N* (Fig. 2*C*) and provides further evidence that the *N*-large condition was accompanied by lower representational uncertainty. According to a linear mixed-effect model analysis (LMM5), the logarithm of RT, log(RT), increased with *p*(1 – *p*) (*F*_(1,22.35)_ = 50.34, *P* < 0.001), but decreased with *N* (*F*_(1,26.16)_ = 34.45, *P* < 0.001) and *p* (*F*_(1,25.95)_ = 7.00, *P* = 0.014).

In sum, we found that a considerable portion of variability in subjects' probability distortion functions and RT patterns can be accounted by differences in representational uncertainty. One may wonder whether the effects of representational uncertainty can be explained away by task difficulty. We doubt not, because higher representational uncertainty does not necessarily correspond to higher task difficulty. For example, representational uncertainty for relative frequency estimation is proportional to *p*(1 – *p*), which is maximal at *p* = 0.5 and minimal when *p* is close to 0 or 1. In contrast, regarding difficulty, there seems to be little reason to expect that relative frequency estimation itself should be more difficult for *p* = 1/2 than for *p* = 1/3, or be more difficult for *p* = 1/3 than for *p* = 1/4. As another counterexample, representational uncertainty modeled in the present study is higher in the *N*-small condition than in the *N*-large condition (Fig. 2*C*), but there seems to be little reason to expect relative frequency estimation to be more difficult for displays with fewer dots. It is only when the brain tries to compensate for potential sampling errors in the estimation that it may find more uncertainty (but not difficulty) in representing probability values closer to 0.5 than representing those closer to 0 or 1, and likewise different levels of uncertainty for different numerosities of dots (Zhang et al., 2020).

### Neural entrainment to periodic changes of *p* or *p*(1 – *p*)

After showing that the BLO models can well capture the behavioral results, we next examined whether the brain response could track the periodically changing *p* values (*P*-cycle) or *p*(1 – *p*) values (*U*-cycle) in the stimulus sequence. Recall that the *P*-cycle and *U*-cycle trials had identical individual displays and differed only in the ordering of the displays: In *P*-cycle trials, *p* alternated between small and large values in cycles of 3.33 Hz while *p*(1 – *p*) was aperiodic; in *U*-cycle trials, *p*(1 – *p*) alternated in cycles of 3.33 Hz while *p* was aperiodic.

**Figure 4.**
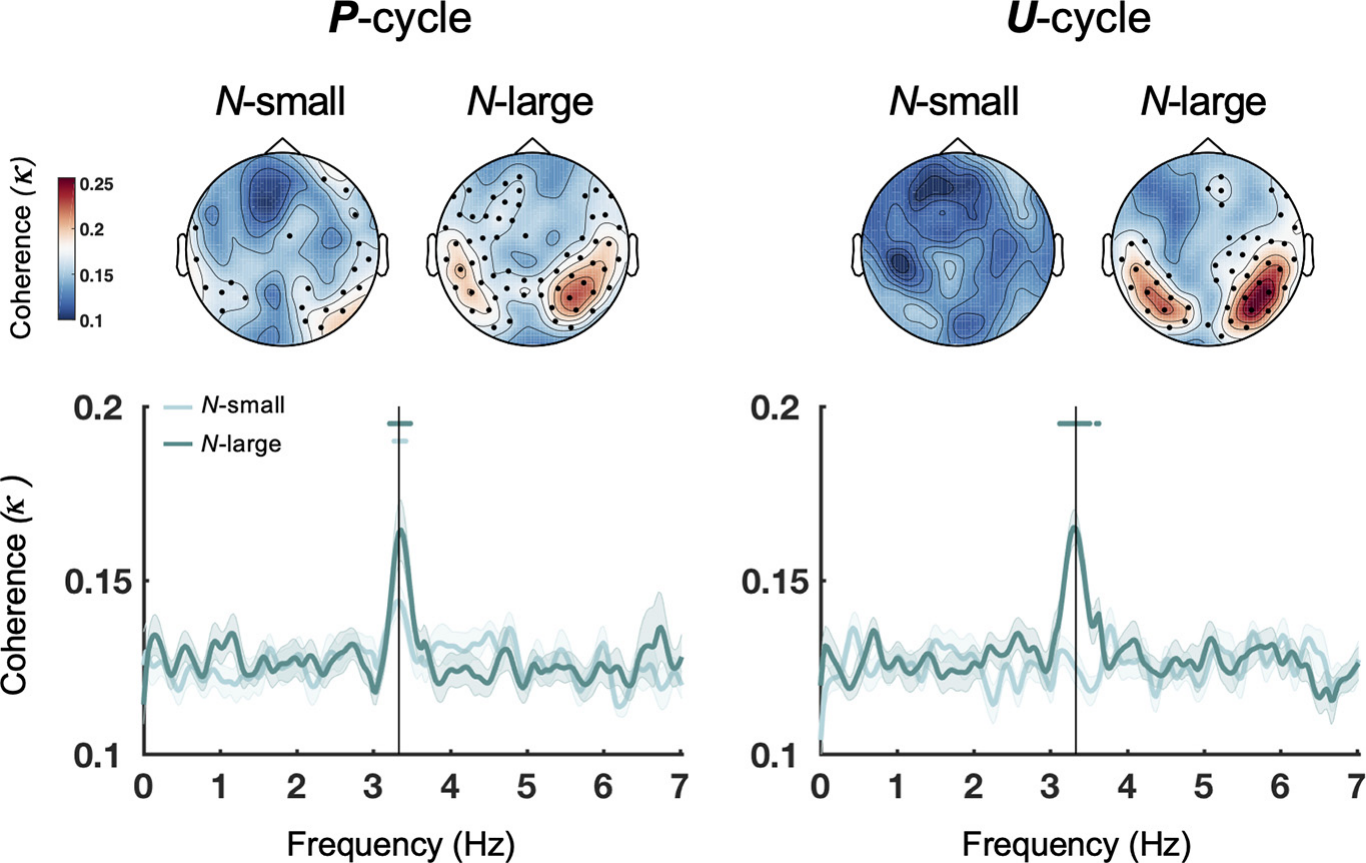
Results of phase coherence analysis. Grand-averaged phase coherence spectrum for magnetometers. Left, The phase coherence (κ) between the periodic *p* values and MEG time series in *P*-cycle trials. Right, The phase coherence between the periodic *p*(1 – *p*) values and MEG time series in *U*-cycle trials. Light and dark green curves indicate the *N*-small and *N*-large conditions, respectively. Shadings represent SEM across subjects. Vertical line indicates 3.33 Hz. Dots above the spectrum represent the frequency bins whose phase coherence was significantly above chance level (permutation tests, *P_FDR_* < 0.05 with *FDR* correction across frequency bins). Insets, Grand-averaged phase coherence topography at 3.33 Hz for each cycle and numerosity condition. Solid black dots represent sensors whose phase coherence at 3.33 Hz was significantly above chance level (permutation tests, *P_FDR_* < 0.05 with *FDR*-corrected across magnetometers).

As shown in Figure 4, the brain response indeed tracked the periodic changes of *p* and *p*(1 – *p*). In particular, in *P*-cycle trials (Fig. 4, left), the phase coherence between the periodic *p* values and the MEG time series reached significance at 3.33 Hz (permutation test, *FDR*-corrected *P_FDR_* < 0.05), mainly in the occipital and parietal sensors. Importantly, significant phase coherence was also found at 3.33 Hz in *U*-cycle trials between the periodic *p*(1 – *p*) values and the MEG time series (Fig. 4, right). That is, the brain activity was also entrained to the periodic changes of the representational uncertainty of *p* (i.e., *p*(1 – *p*)). Given that subjects were only asked to track the value of *p* but not *p*(1 – *p*), the observed neural entrainment to the task-irrelevant *p*(1 – *p*) suggests an automatic encoding of representational uncertainty in the brain.

Notably, the significant phase coherences between the periodic *p* or *p*(1 – *p*) values and MEG time series were not simply because the MEG time series consisted of 3.33 Hz frequency components. In a control analysis, we calculated the phase coherence between the aperiodic variable (i.e., *p* in *U*-cycle trials or *p*(1 – *p*) in *P*-cycle trials) and the same MEG time series, and found that there were no significant peaks at 3.33 Hz. Moreover, we had carefully controlled potential confounding variables in our experimental design. For example, *p* and *p*(1 – *p*) were linearly independent of each other, both of which had negligible correlations with numerosity or low-level visual features, such as luminance, contrast, and color variance (for details, see Materials and Methods). Furthermore, different from *p* and *p*(1 – *p*), these potential confounding variables were associated with similar entrainment responses in *P*-cycles and *U*-cycles.

### Behavioral probability distortion and the neural entrainment to *p* and *p*(1 – *p*)

We next examined the relationship between behavioral probability distortion and the neural entrainment to *p* and *p*(1 – *p*) on a subject-by-subject basis. Specifically, the phase coherence between a specific variable (*p* or *p*(1 – *p*)) and the MEG time series can be considered as a measure of the strength of neural responses to the variable (Kramer and Eden, 2016). We defined the following:

(14) $$\beta =\frac{\kappa_{p}}{\kappa_{p\left(1-p\right)}}$$

to quantify the relative strength of *p* to *p*(1 – *p*) in neural responses, where $\kappa_{p}$ denotes the phase coherence for *p* in *P*-cycle trials, and $\kappa_{p\left(1-p\right)}$ denotes the phase coherence for *p*(1 – *p*) in *U*-cycle trials. A higher value of β would imply a stronger neural encoding of relative frequency or a weaker encoding of representational uncertainty and is thus supposed to yield probability distortions of a greater slope (Zhang et al., 2020). Both the behavioral and neural measures we defined below were intrasubject ratios that were unitless and scale-free, thus not subject to the potential scaling issues in interindividual correlation (Lebreton et al., 2019).

Given that the estimated slope of distortion $\hat{\gamma }$ was greater in *N*-large trials than in *N*-small trials, we would expect β to change in the same direction across the numerosity conditions. That is, suppose we define $\Delta \hat{\gamma }=\ln\left(\frac{{\hat{\gamma }}_{N\text{-}\mathrm{large}}}{{\hat{\gamma }}_{N\text{-}\mathrm{small}}}\right)$ and $\Delta \beta =\ln\left(\frac{\beta_{N\text{-}\mathrm{arge}}}{\beta_{N\text{-}\mathrm{small}}}\right)$, there should be a positive correlation between $\Delta \hat{\gamma }$ and Δβ.

**Figure 5.**
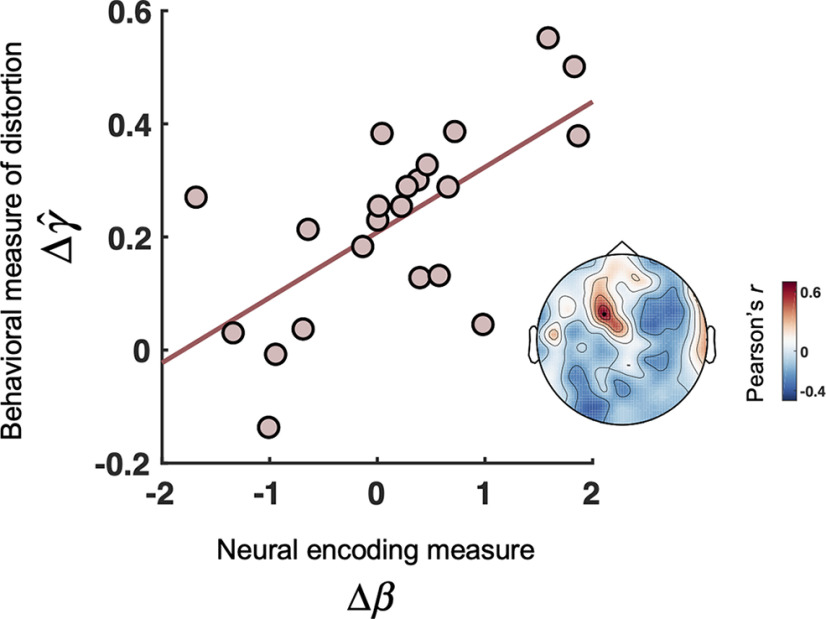
Neural responses to *p* and *p*(1 – *p*) predict the slope of probability distortion. We defined $\Delta \hat{\gamma }=\ln\left(\frac{{\hat{\gamma }}_{N\text{-}\mathrm{large}}}{{\hat{\gamma }}_{N\text{-}\mathrm{small}}}\right)$ and $\Delta \beta =\ln\left(\frac{\beta_{N\text{-}\mathrm{large}}}{\beta_{N\text{-}\mathrm{small}}}\right)$ to quantify, respectively, how much the behavioral measure of the slope of distortion, $\hat{\gamma }$, and the relative strength of *p* to *p*(1 – *p*) in neural responses, β, changed across the two numerosity conditions. Main plot, Correlation between $\Delta \hat{\gamma }$ and the Δβ at the frontoparietal magnetometer channel MEG0421 (Pearson's *r* = 0.67, one-tailed *P_FDR_* = 0.033 with *FDR* correction across 102 magnetometers). The logarithm transformation was used only for visualization. Each dot represents 1 subject. Inset, Correlation coefficient topography between $\Delta \hat{\gamma }$ and Δβ, on which MEG0421 is marked by a solid black dot.

As shown in Figure 5, there was indeed a significant correlation between behavioral and neural measures across subjects in the frontoparietal region (Pearson's *r* = 0.67, *FDR*-corrected one-tailed *P_FDR_* = 0.033), associating the neural entrainment to *p* and *p*(1 – *p*) with probability distortion behaviors.

### Neural encoding of *p* and *p*(1 – *p*) over time and the associated brain regions

After establishing the automatic tracking of cyclic changes in *p*(1 – *p*) and its behavioral relevance, we next aimed to delineate the temporal dynamics of *p* and *p*(1 – *p*) in the brain signals. Recall that on each trial one of the two variables of our interest (*p* or *p*(1 – *p*)) changed periodically and the other was aperiodic. Therefore, we could decode the temporal course of aperiodic *p*(1 – *p*) in *P*-cycle trials and aperiodic *p* in *U*-cycle trials.

In particular, we performed a time-resolved decoding analysis based on all 306 sensors (see Materials and Methods) using a regression approach that has been used in previous EEG and MEG studies (Wyart et al., 2012; Crosse et al., 2016), including ours (Jia et al., 2017; Liu et al., 2017; Huang et al., 2018). The intuition of the time-resolved decoding analysis is as follows. Suppose the onset of each new *p* or *p*(1 – *p*) value in the stimulus sequence evokes phase-locked brain responses that extend across time and whose magnitudes are proportional to the encoded value. The resulting MEG time series would then be a superposition of the responses to all the preceding stimuli in the sequence. But as soon as the values of an encoded variable are not correlated over time (i.e., free of autocorrelation), their brain response profiles are separable. In particular, for any stimulus in the sequence, we can use the MEG signals at a specific delay after the stimulus onset to predict the value of the stimulus. Importantly, different from the phase coherence analysis (Fig. 4), which only reflects the overall strength of the brain responses, this time-resolved decoding analysis allows us to assess how *p* and *p*(1 – *p*) are encoded over time.

**Figure 6.**
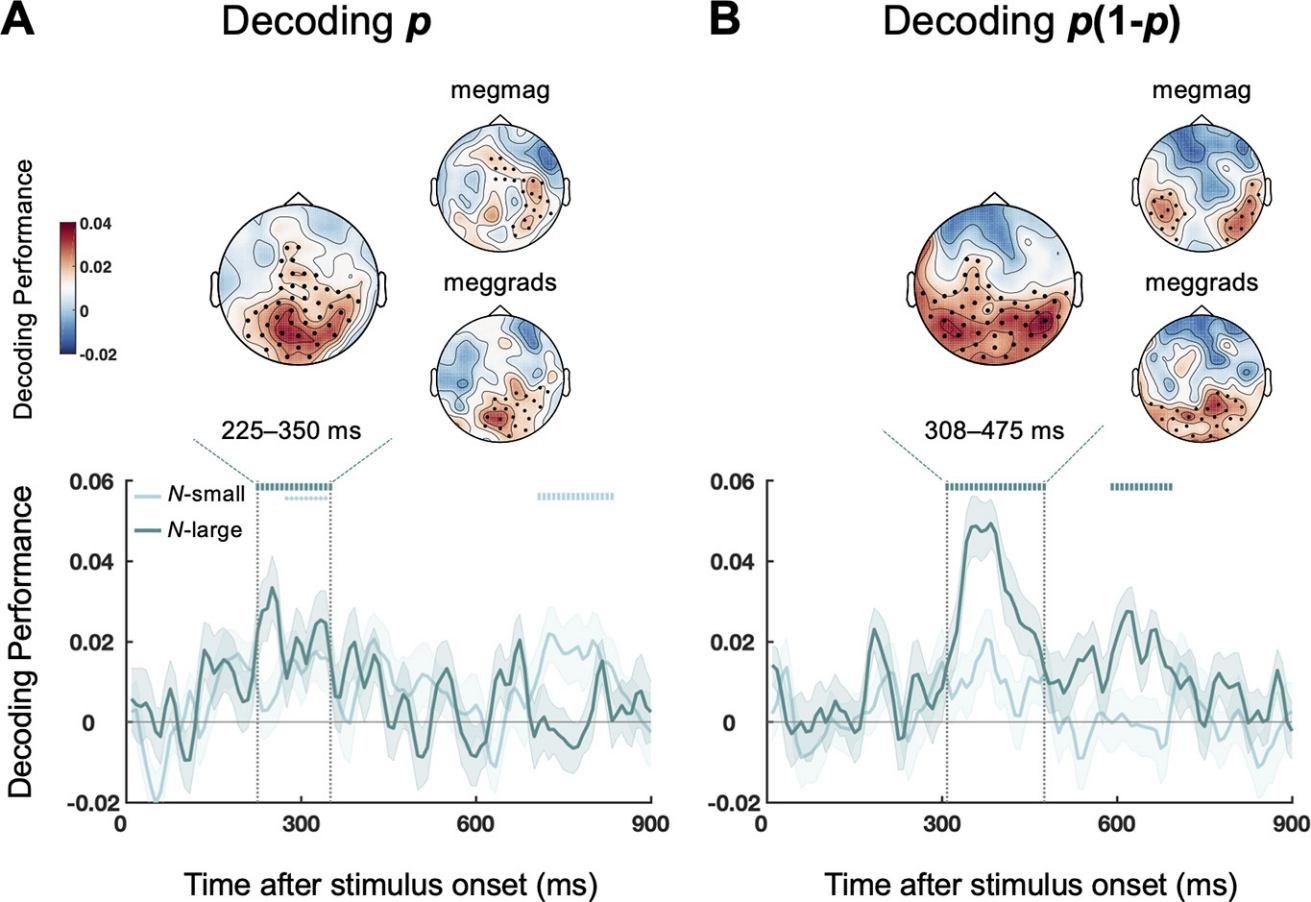
Results of decoding analyses. Main plots, Time-resolved decoding performance over different time lags for *p* (***A***) and *p*(1 – *p*) (***B***), separately for the *N*-small (light green) and *N*-large (dark green) conditions. Shadings represent SEM across subjects. Symbols above the curves represent time lags that had above-chance decoding performance (cluster-based permutation tests). Vertical bars represent *P_cluster_* < 0.01. Dots represent 0.01 ≤ *P_cluster_* < 0.05. Insets, Topography of spatial decoding performance for the time window (highlighted by the funnel-shaped dashed lines) that contained the peak decoding performance in the time-resolved decoding analysis. Larger plot of topography represents the spatial decoding results using temporal samples from both magnetometers and gradiometers at each location. Smaller plots of topography represent the spatial decoding results separately for magnetometers (megmag) and pairs of gradiometers (meggrads). Solid black dots represent the sensor clusters with above-chance decoding performance (*P_cluster_* < 0.05, corrected for multiple comparisons using cluster-based permutation test) for the *N*-large condition. The time-resolved decoding performance for *p* peaked at ∼327 ms after stimulus onset, whereas that for *p*(1 – *p*) peaked at ∼419 ms. The encodings of *p* and *p*(1 – *p*) involved overlapping parietal-occipital regions, including the frontoparietal region where Δβ and $\Delta \hat{\gamma }$ were positively correlated (Fig. 5).

Figure 6 plots the temporal course of the decoding performance for *p* and *p*(1 – *p*). We found that both *p* and *p*(1 – *p*) were successfully decoded from the MEG signals in *N*-large trials (cluster-based permutation test, *P_cluster_* < 0.05). In particular, the decoding performance for *p* peaked ∼327 ms after stimulus onset (Fig. 6*A*), whereas that for *p*(1 – *p*) peaked ∼419 ms after stimulus onset (Fig. 6*B*). In other words, the neural encodings of relative frequency and its representational uncertainty had distinct time courses, with the latter occurring ∼100 ms later than the former (95% CI: 17-194 ms).

As a confirmation of our decoding results from aperiodic sequences, we performed additional time-resolved decoding analysis for periodic stimulus sequences, that is, *p* in *P*-cycle trials and *p*(1 – *p*) in *U*-cycle trials. Despite that the periodicity of the stimulus sequence prevented us from separating the neural responses at time *t* from *t* + 150 ms, *t* + 300 ms, or *t* + 450 ms, etc, the largest peaks decoded from these periodic sequences fell into the same time window as those of the aperiodic sequences (i.e., 225-350 ms for *p* and 308-475 ms for *p*(1 – *p*)).

**Figure 7.**
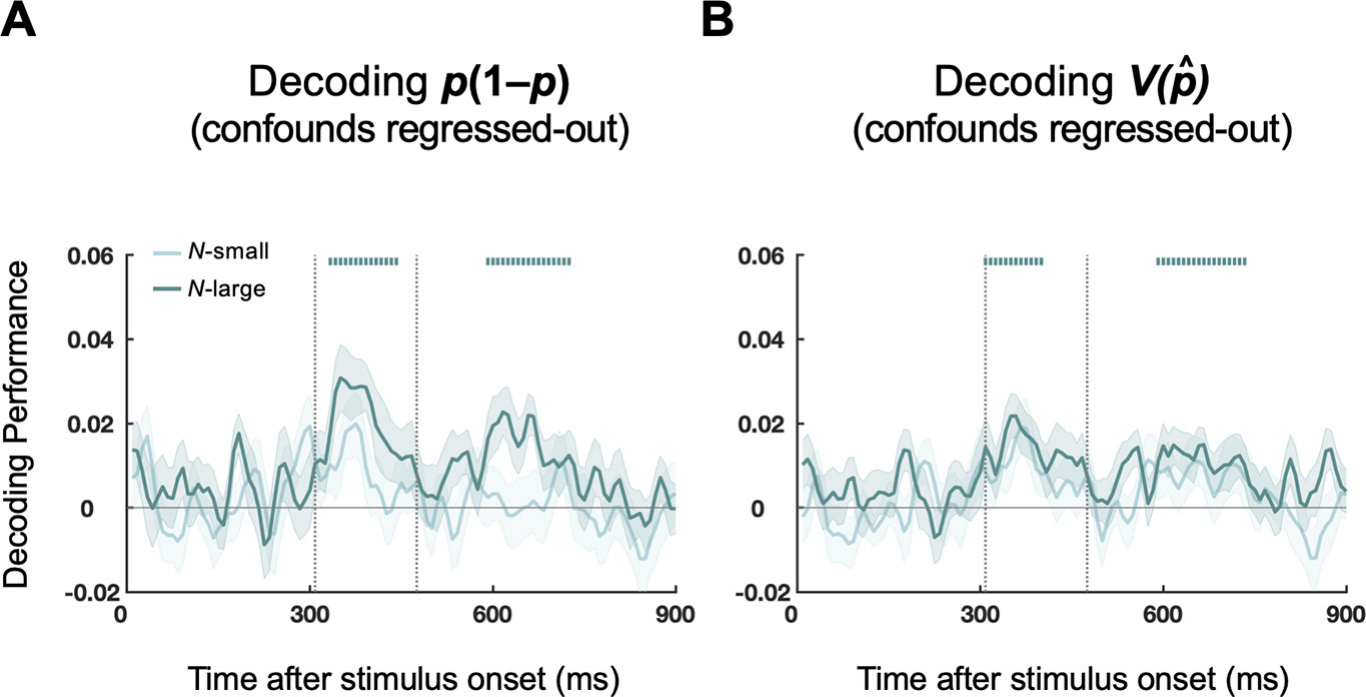
Time-resolved decoding analysis based on CVCR. ***A***, Decoding performance for *p*(1 – *p*) with confounds regressed-out. ***B***, Decoding performance for $V\left(\hat{p}\right)$ with confounds regressed-out. The $V\left(\hat{p}\right)$ was computed according to each subject's fitted BLO model. Curves indicate grand-averaged decoding performance over different time lags, separately for the *N*-small (light green) and *N*-large (dark green) conditions. Shadings represent SEM across subjects. Vertical bars above the curves represent time lags that had above-chance decoding performance (cluster-based permutation tests, *P_cluster_* < 0.01). Eight confounding variables (including *N*) were regressed out from MEG time series before decoding analysis (for methodological details, see Materials and Methods). The decoded time course from CVCR for *p*(1 – *p*) was similar to that of the standard time-resolved decoding analysis (see Fig. 6*B*). The decoded time course was also similar for $V\left(\hat{p}\right)$.

In contrast, none of the potential confounding variables (numerosity, luminance, etc.) showed temporal courses similar to *p* or *p*(1 – *p*) when the same time-resolved decoding procedure was applied. The automatic encoding of the task-irrelevant *p*(1 – *p*) might seem surprising. To further verify that this was not an artifact of potential low-level confounds, we performed an additional decoding analysis for *p*(1 – *p*) based on the CVCR (Snoek et al., 2019), where the confounding variables were regressed out from MEG time series before time-resolved decoding analysis (for details, see Materials and Methods). The decoded temporal course for *p*(1 – *p*) was little changed (Fig. 7*A*). A similar decoding analysis for the complete form of representational uncertainty $V\left(\hat{p}\right)$ resulted in similar temporal course as that of *p*(1 – *p*) (Fig. 7*B*). In sum, we found automatic encoding of *p*(1 – *p*) in brain signals even when it was task-irrelevant and when low-level confounding factors were excluded.

Further, based on the time windows that achieved the highest time-resolved decoding performance, we performed spatial decoding analyses at each sensor location separately for *p* and *p*(1 – *p*) to assess the associated brain regions (see Materials and Methods). The topographies using magnetometers, pairs of gradiometers, or both of them are shown in Figure 6. We found that the encoding of the two variables involved overlapping parietal-occipital regions, including the frontoparietal region where Δβ and $\Delta \hat{\gamma }$ were positively correlated (Fig. 5).

## Discussion

We used MEG to investigate probability distortions in a relative frequency estimation task and found that the human brain encodes not only the task-relevant relative frequency but also its task-irrelevant representational uncertainty. The neural encoding of the representational uncertainty occurs at as early as ∼400 ms after stimulus onset. These results suggest that the human brain automatically and quickly quantifies the uncertainty inherent in probability information. Our findings provide neural evidence for the theoretical hypothesis that probability distortion is related to representational uncertainty. More generally, these findings may connect to the functional role of confidence (estimation of uncertainty) during judgment and decision-making.

### Neural computations underlying probability distortions

Humans show highly similar probability distortions in tasks involving probability or relative frequency, with the subjective probability typically an inverted-*S*-shaped function of the objective probability (Zhang and Maloney, 2012). Why do people distort probability information in such a systematic way? Though inverted-*S*-shaped distortions of probability had been found in the activity of a few brain regions (Tobler et al., 2008; Hsu et al., 2009), the neural computations involved in transforming objective probabilities to subjective probabilities were largely unknown. Several theories (Martins, 2006; See et al., 2006; Fennell and Baddeley, 2012; Zhang et al., 2020) rationalize probability distortion as a consequence of compensating for representational uncertainty, in accordance with the framework of Bayesian Decision Theory (Maloney and Zhang, 2010). In brief, the brain will discount an internal representation of probability according to the level of uncertainty so that it can achieve a more reliable estimate of the objective probability. The higher the uncertainty associated with a representation, to a greater extent the representation is discounted. By modeling human subjects' probability distortion behavior in two representative tasks, relative frequency estimation and decision under risk, Zhang et al. (2020) found evidence that representational uncertainty proportional to *p*(1 – *p*) is compensated in human representation of probability or relative frequency.

What we found in the present study is neural evidence for the encoding of *p*(1 – *p*) during the evaluation of *p*. For the first time, we have shown that representational uncertainty *p*(1 – *p*) is not just virtual quantities assumed in computational models of probability distortions but is really computed in the brain.

### Representational uncertainty and confidence

The representational uncertainty that concerns us lies in the internal representation of probability or relative frequency. One concept that may occasionally coincide with such epistemological uncertainty but is entirely different is outcome uncertainty, which is widely studied in the literature of decision under risk (Fiorillo et al., 2003; Tobler et al., 2007), prediction error (Fiorillo et al., 2003), and surprise (Preuschoff et al., 2011; van Lieshout et al., 2018). For example, for a two-outcome gamble with probability *p* to receive *x_1_* and probability 1 – *p* to receive *x_2_*, the variance of the outcomes is proportional to *p*(1 – *p*). In the present study, the relative frequency estimation task we chose to investigate does not involve similar binary uncertain outcomes and thus avoids the possible confusion of outcome uncertainty with representational uncertainty. Therefore, the neural encoding of *p*(1 – *p*) we found here cannot be attributed to the perception of outcome uncertainty.

The latency and spatial topography of the neural encoding of representational uncertainty reported here may remind the reader of the P300 ERP component, a well-known neural marker of surprise (Sutton et al., 1965; Donchin, 1981; Mars et al., 2008); that is, more rare and thus more surprising events in a stimulus sequence will evoke a stronger positive ERP component that peaks ∼300 ms after stimulus onset. In the present study, however, the task-relevant stimulus value *p* was uniformly sampled between 0.1 and 0.9, which implies that no events in our stimulus sequences could be considered more rare or surprising than any other events. Therefore, the encoding of representational uncertainty we found at ∼400 ms should not derive from P300.

Another concept that may sound similar to but is actually different from representational uncertainty is task difficulty. Conceptually, representational uncertainty is closely related to the uncertainty of estimation because of random sampling errors (Zhang et al., 2020), whereas task difficulty is usually associated with cognitive demands or efforts (Philiastides et al., 2006; Bankó et al., 2011). As we reasoned earlier, *p*(1 – *p*) is positively correlated with the representational uncertainty of *p*, but not necessarily with task difficulty. Moreover, previous EEG or MEG studies on task difficulty did not report the fast phasic neural responses that we found here for encoding representational uncertainty (peaking at ∼400 ms). In particular, though a negative ERP component as early as ∼220 ms whose amplitude increased with the level of visual noise in an image classification task was once identified as a neural signature of decision difficulty (Philiastides et al., 2006), it was later found to be a specific neural response to visual noise level instead of to difficulty in general (Bankó et al., 2011).

Indeed, representational uncertainty is more closely related to confidence, except that it is not necessarily bound to any specific judgment or decision (Pouget et al., 2016). Specifically, the encoding of representational uncertainty may be understood as a special case of the “second-order valuation” proposed by Lebreton et al. (2015). As described previously, Lebreton et al. (2015) found a principled relationship between an overt numerical judgment and the individual's confidence about the judgment, with the latter being a quadratic function of the former. They showed in fMRI studies that such quadratic form, as a proxy to confidence, is automatically encoded in ventromedial PFC, even when confidence rating is not explicitly required. Using the same quadratic form as the proxy, here we found automatic encoding of the representational uncertainty of relative frequency during the tracking of a visual sequence. Meanwhile, our findings extend previous work and contribute to the confidence literature in the following three aspects.

1. We found automatic encoding of representational uncertainty even in the absence of overt judgment, not just in the absence of overt confidence estimation. In our experiment, subjects were asked to track the relative frequency in the stimulus sequence but made no overt judgment, except for the last display. Moreover, displays were refreshed every 150 ms, apparently leaving no time for deliberation.
2. We resolved the temporal course of the encoding of representational uncertainty, which would be inaccessible under the low temporal resolution of fMRI. In particular, representational uncertainty is encoded as early as 400 ms on stimulus onset, ∼100 ms after the encoding of relative frequency itself. The parietal-occipital region is involved in the processing of both relative frequency and its representational uncertainty but during different time windows. The fast encoding of representational uncertainty we found echoes recent psychophysiological findings in humans (Zizlsperger et al., 2014; Gherman and Philiastides, 2015, 2018) and nonhuman primates (Kiani and Shadlen, 2009) that the confidence encoding for perceptual judgments can be detected in the brain far before confidence rating and even before the overt judgment or decision-making.
3. Our findings connect to the functional importance of confidence encoding in information processing (Bahrami et al., 2010; Koriat, 2015; Pouget et al., 2016) that is still not well understood but receives growing attention. In particular, here we ask how the neural encoding of representational uncertainty may relate to probability distortion. By comparing experimental conditions under which the same individuals' distortions of relative frequency differed, we found that a relatively stronger response to representational uncertainty in the frontoparietal region corresponds to a shallower slope of distortion. We conjecture that the automatic, fast encoding of representational uncertainty might not just be *post hoc* evaluation but indeed regulate probability distortion. Meanwhile, we are aware that our current findings are based on correlational analyses, and whether there is causality between the neural encoding of representational uncertainty and probability distortion still awaits future empirical tests.

### Methodology implications

The SSR technique (Norcia et al., 2015), using rapid periodic stimulus sequence to entrain brain responses, has been widely used with EEG/MEG to investigate low-level perceptual processes, which increases the signal-to-noise ratio of detecting the automatic brain responses to the stimuli by sacrificing temporal information. In our experiment, we constructed periodic *p* or *p*(1 – *p*) sequences and showed that SSR can also be used to reveal the brain's automatic responses to these more abstract variables. Moreover, we demonstrate the feasibility to perform time-resolved decoding for the other aperiodic variable embedded in the same sequence, thus exploiting the advantages of both the SSR and time-resolved decoding techniques. Such design would be useful for a broad range of problems that need to dissociate the processing of two or more variables in brain activities.
